# Opinionated views on biophysical and social constraints on agroforestry system

**DOI:** 10.3389/fpls.2025.1607207

**Published:** 2025-08-14

**Authors:** Xinjie Zha, Zhijie Zhang

**Affiliations:** ^1^ Xi’an University of Finance and Economics, Xi’an, China; ^2^ Institute of Strategic Planning, Chinese Academy of Environmental Planning, Beijing, China

**Keywords:** agroforestry, biophysical constraints, climate change, resource competition, ecosystem services

## Introduction

Agroforestry is a collective concept that integrates indigenous, traditional and modern land-use systems combining tree cultivation with agricultural crop production and/or animal husbandry, including alley cropping, windbreaks, and silvopasture (Eichhorn et al., 2006; Terasaki Hart et al., 2023). By the late 1970s, growing awareness of the environmental and social consequences of intensified agricultural systems following the Green Revolution led to the rising recognition of agroforestry as a viable nature-based solution (NbS) and a sustainable alternative to conventional monoculture farming. Agroforestry systems enhance resource efficiency by strategically integrating species with complementary ecological niches, optimizing spatial, temporal, and physical resource use. This strategy boosts productivity in both food (e.g., cereals, vegetables and woody crops) and non-food outputs, such as timber, bioenergy, and other biomass-based materials (Dalemans et al., 2019). Furthermore, agroforestry enhances carbon sequestration or protects carbon storage on agricultural lands, with a cost-effective mitigation potential estimated between 0.12 Pg C yr^−1^ (Griscom et al., 2017) and 0.31 Pg C yr^−1^ (Roe et al., 2021). This potential estimated is comparable to other key strategies, such as reforestation (0.27 Pg C yr^−1^) and reducing deforestation (0.49 Pg C yr^−1^).

**Figure 1:**
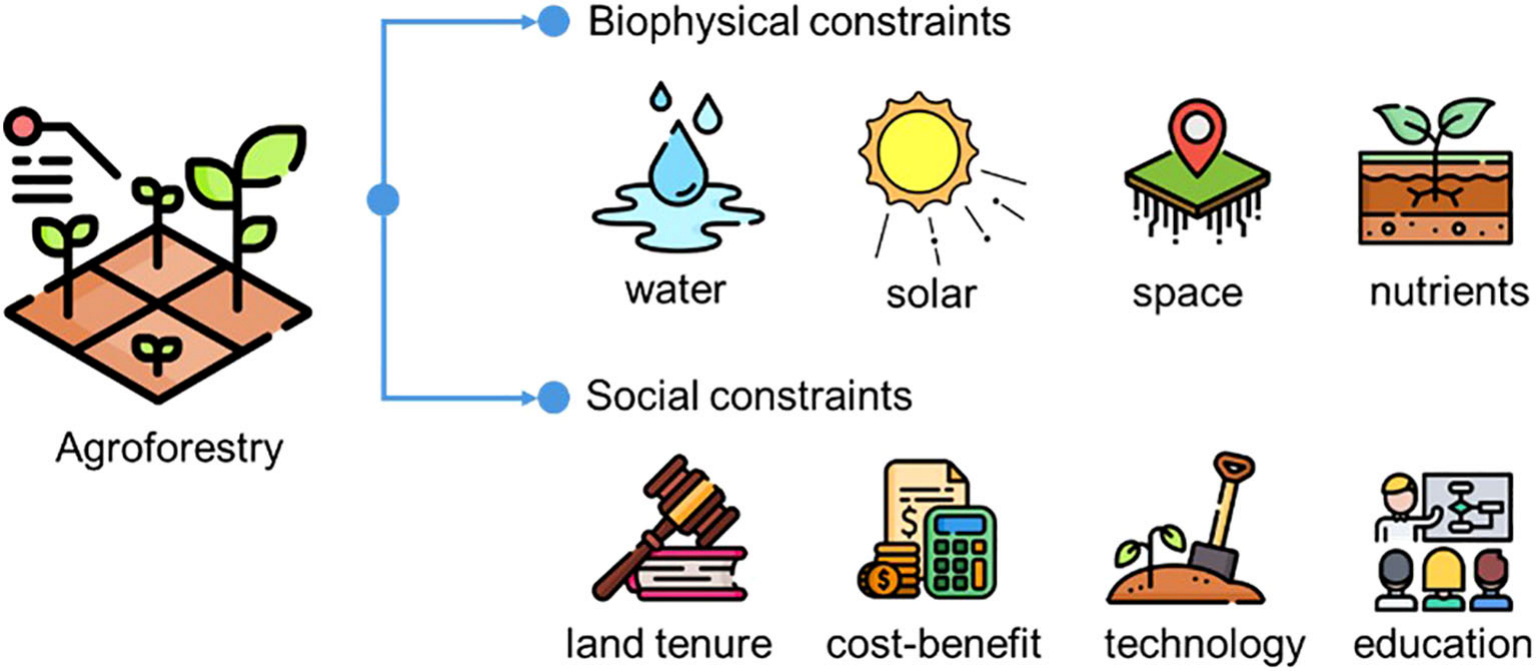
Biophysical and social constraints on agroforestry system. Icons sourced from Flaticon (www.flaticon.com).

The overarching advantage of agroforestry lies in its multifunctionality: enhance biodiversity, mitigates climate change, reduces land degradation, improve soil health and strengthen food security and dietary diversity, while supporting sustainable livelihoods (Torralba et al., 2016; Beillouin et al., 2021). Although the ecological performance still lagged behind that of natural forest [11% lower biodiversity and 37% lower ecosystem services sourced from De Beenhouwer et al. (2013)], yet substantially higher than conventional monoculture agriculture. A meta-analysis synthesizing 365 comparisons (Torralba et al., 2016) demonstrated a significant positive effect of agroforestry on ecosystem services supply, including food production (+17.3%), soil fertility (+26.1%), biodiversity (+29.7%), and erosion control (+223%). Among agroforestry types, silvopastoral systems showed a lower mean effect size (0.324) is less than silvoarable (0.772). It is highlighted as one of the most effective options to address the multiple environmental issues and social crisis (IPCC, 2019). Currently, about 43% of agricultural land under some variation of agroforestry approaches (Zomer et al., 2016), and approximately 1.8 billion people directly or indirectly depend on agroforestry products and services for their livelihood (ICRAF, 2006). The adoption of agroforestry remains uneven across regions: India alone manages approximately 28 Mha under agroforestry systems (Food and Agriculture Organization of the United Nations (FAO), 2021), while the EU has around 15 Mha, primarily in the form of Mediterranean wood-pasture landscapes (den Herder et al., 2016). This number is expected to rise, as agroforestry continues to receive policy incentives and supportive subsides from various agriculture- and forestry-related frameworks, such as the Common Agricultural Policy (CAP) by the European Union (EU), the Farm Bill by the United States, and AFR100 (African Forest Landscape Restoration Initiative). For example, EU’s CAP offers farmers €60–120/ha/year for maintaining tree–crop systems and €100–350/ha/year for ongoing management, with some programs covering up to 100% of establishment costs. In the Amazon region, agroforestry projects promote coffee intercropped with native tree species or diverse tree mixtures, aiming to support smallholder livelihoods while conserving forests. The EverGreen Agriculture Partnership advances the systematic integration of trees into agricultural landscapes across sub-Saharan Africa, with a particular emphasis on nitrogen-fixing species to restore soil fertility and boost agricultural productivity. However, despite its potential benefits, the widespread adoption and expansion of agroforestry systems remain hindered by several biophysical and social constraints, necessitating systematically summarized and targeted interventions to fully unlock the benefits of agroforestry (**Figure 1**).

## Biophysical constraints on agroforestry

Biophysical constraints refer to the inherent physical and biological limitations of natural systems that may affect the feasibility, effectiveness (ecosystem service and productive functions), or long-term sustainability of agroforestry systems. While artificial inputs and management interventions can often overcome these barriers, adoption ultimately hinges on whether the practice is cost-effective—both economically and ecologically. Similar to all living organisms, the biological components of agroforestry systems—including annual and perennial plants, as well as livestock—will be increasingly exposed to rising temperatures, elevated atmospheric CO_2_ levels, and shifting precipitation patterns (Burke et al., 2009). Climate change-driven shifts in temperature and precipitation patterns (e.g., extreme droughts, irregular rainfall, seasonal droughts) can alter the growth (e.g., flowering, pollination, and even plant mortality) and suitability of tree species and crops cultivated in agroforestry, but highly depend on emission scenarios and geographic location (Odeny et al., 2019; Ivezić et al., 2021; Lima et al., 2022). Elevated temperatures increase evapotranspiration, resulting in soil moisture loss and plant heat stress. Many agroforestry species exhibit thermal thresholds, beyond which photosynthetic efficiency declines, flowering phenology shifts, fruit set and crop yield decreases (Guillot et al., 2019). Furthermore. niche modeling predictions indicate that, under various scenarios of future climate change, the suitable habitat for 135 tree species traditionally cultivated in Brazilian agroforestry systems is projected to decline by 22.3% to 56.3% (Lima et al., 2022). Similarly, in Tanzania, different agroforestry systems (e.g., *Albizia gummifera*, *Persea americana* and *Mangifera indica*) are expected to exhibit varying movement responses (upslope or downslope migration) to climate change (Odeny et al., 2019).

The competition between trees and crops for resources of space, water, sunlight and soil nutrition is critical determinant constraining agroforestry productivity and monetary revenue (Swieter et al., 2022). In water-limited and arid environments, trees and crops often compete for limited water resources and thus reduce yields. Such competition is particularly pronounced when deep-rooted trees extract water from underground reserves, potentially depleting groundwater supplies and exacerbating water scarcity. Tree shading can positively impact crop yield by modifying the microclimate, reducing evapotranspiration, and facilitating hydraulic lift—where plant roots transfer water from moist to drier soil layers, enhancing soil moisture retention (Bayala and Prieto, 2019). However, factors such as tree height, canopy density, and orientation regulate the extent of solar radiation reaching the cropped area throughout the day (Schmidt et al., 2019). It is produced in high-latitude regions, where the growing season is already shortened by limited daylight hours, and the lower angle of sunlight further restricts light penetration (van der Werf et al., 2007). In silvopastoral systems, resource competition between trees, pasture, and livestock is also prominent. This includes root-level competition for water and nutrients, excessive canopy shading that suppresses pasture growth, and livestock browsing that damages young trees and hinders their establishment (Jose and Dollinger, 2019; Karki et al., 2019). Rivest et al. (2013) highlight the lack of consensus on how trees influence annual crop yields, affected by depending on tree functional groups (e.g., deciduous, evergreen oak) and rainfall conditions. Such resource competition happened in aboveground and belowground part in agroforestry systems (Zhang et al., 2013; Isaac et al., 2014). For example, nutrient competition in a walnut tree root and wheat system caused nutrient depletion, negatively affecting plant health and agricultural productivity (Zhang et al., 2013). Soil fertility management, including mulching, composting, and nitrogen-fixing tree species, is necessary to mitigate these challenges.

Integrating trees into agricultural systems can influence pest and disease dynamics in complex ways, which are context-dependent, including species composition, landscape configuration and management practices. On the one hand, agroforestry systems can also suppress pest outbreaks by enhancing habitat for natural enemies, increasing plant diversity, and disrupting pest life cycles (Pumariño et al., 2015; Gurr et al., 2017). On the other hand, the interactions of tree-crop in agroforestry systems can create microclimates that favor pest proliferation, particularly in humid environments where fungal diseases thrive. This risk is especially pronounced in monoculture tree plantations within agroforestry systems (Ambele et al., 2018), where large-scale uniform tree stands provide continuous food sources and habitat for specific pests. Staton et al. (2021) suggested that agroforestry systems are likely to suppress annual, disturbance-tolerant weeds and highly mobile specialist pests, while perennial weeds and low-mobility generalist pests may persist or even proliferate. This is largely attributed to the greater structural complexity, diverse microclimates, and extended crop cycles inherent to agroforestry, which create less favorable conditions for short-lived, disturbance-adapted species while simultaneously offering more stable habitats for perennial weeds and less mobile pests.

## Social constraints on agroforestry

The primary social barriers limiting the widespread adoption of agroforestry were derived from weak policy support, financial concerns and a lack of knowledge and management capacity. Among the most critical barriers is land tenure insecurity, often proven more important than other motives for agroforestry investments (e.g., cash subsidies), particular in the Global South, where land laws and policies often fail to clearly define ownership rights for trees planted on farmland (McElwee, 2009). Many high-priority areas for restoring conventional farmland through agroforestry overlap significantly with regions characterized by weak governance and unrecognized land rights, posing a challenge to the widespread adoption of agroforestry (Rakotonarivo et al., 2023). In many regions, customary land tenure systems separate land ownership from tree ownership, necessitating alignment between the land-use strategies and interests of both land and tree owners (Folefack and Darr, 2021). The significant disparity between statutory and customary land tenure systems presents fundamental challenges to achieving the intended ecological and social benefits of restoration (McLain et al., 2021). Massive local questionnaire confirmed that farmers with insecure land tenures and agroforestry rights are less motivated to adopt agroforestry (Folefack and Darr, 2021; Jha et al., 2021).

Another major concern is the financial constraints of agroforestry systems, particularly among smallholder farmers and resource-limited communities. High upfront costs, delayed financial returns, and market uncertainties make agroforestry a risky investment. The integration of fruit or timber trees into intensively managed pastures or croplands often leads to initial reductions in crop yields or grazing capacity, resulting in short-term economic losses. Establishing an agroforestry system demands substantial investment in seedlings, fencing, irrigation, and labor, with returns delayed for years. Timber and fruit trees may take 5–20 years to reach commercial viability, creating a prolonged cash flow gap that challenges farmers dependent on annual crop cycles for subsistence. While the diversified production characteristic of agroforestry can reduce the risks associated with monoculture (England et al., 2020; Akter et al., 2022), however, market fluctuations in the value of agroforestry products—timber, fruits, nuts, and medicinal plants—remain unpredictable, complicating long-term financial planning. Some modeling studies suggest that, in the absence of subsidy mechanisms (e.g., carbon incentives for agroforestry), the returns from agroforestry systems may be not profitable than those of monoculture farming.

Countries with widespread hunger and malnutrition—particularly those in sub-Saharan Africa and large developing nations—continue to face pressing food security challenges (Chen et al., 2011). Per capita food demand is steadily increasing, and some of these countries are struggling to address severe regional undernutrition and agricultural mechanization. National agricultural policies largely favor conventional monoculture farming and intensive agricultural production, offering limited integration of agroforestry into mainstream agricultural systems and failing to provide targeted subsidies. The promotion of agroforestry in such contexts may face significant challenges related to national policy objectives. Smallholder producers, in particular, lack access to stable credit and financial support, discouraging investment in farm inputs, participation in land markets and cash crops cultivation, and making long-term commitments agroforestry (Place, 2009). This situation contrasts with that of developed economies in Europe, North America, and Oceania, where agricultural priorities increasingly focus on precision farming and reducing the environmental footprint of food production. However, agroecological transitions in developed economies may come at the cost of increasing the agricultural footprint in other countries. For example, the implementation of the European Green Deal (EGU), which supports agroforestry, diverse agricultural landscapes and other sustainable land-use practices, could result in an expansion of agricultural land outside the EU by up to 24 Mha, associated with 758.9 Mt CO_2_ emissions and substantial biodiversity loss (Zhong et al., 2024).

From the perspective of NbS, agroforestry’s climate mitigation and ecosystem services’ benefits are largely a public good rather than a private benefit for farmers, leading to market failure (Bettles et al., 2021). Carbon markets and other payment for ecosystem services can regulate this issue by transforming public benefits into private incentives, encouraging greater agroforestry adoption. However, the cost-benefit carbon pricing is estimated at $100/Mg CO_2_, making it challenging for developing countries to set competitive carbon prices (Zeng et al., 2020). Higher-yielding cropland offers the greater per-hectare carbon sequestration potential, but the opportunity cost of agriculture can be substantial, thus requiring careful trade-offs in land-use decisions (Grass et al., 2020; Wurz et al., 2022).

Additionally, the complexity of agroforestry—requiring a deep understanding of tree-crop-livestock interactions, soil management, and market dynamics—can deter farmers from transitioning to more sustainable agroforestry practice. For example, compared to annual crops, tree crops require more complex management, have a longer return period, and pose greater challenges for mechanized production. In silvopastoral systems, careful management of tree species, height, and planting density is essential to minimize resource competition between trees and pasture, and livestock browsing on economically valuable tree species (Smith et al., 2022). Limited access to training programs, extension services, and technical support further restricts their ability to adopt and effectively implement these systems, reinforcing dependence on conventional farming methods.

## Agroforestry systems located at crossroads

The future of agroforestry ultimately depends on whether individual farmers adopt and sustain agroforestry practices, which, in turn, is influenced by the performance of agroforestry systems and the ability of local decision-making environments to minimize the above-mentioned barriers. The performance of agroforestry on productivity and ecosystem services is shaped by the interactions between trees, crops, environment, management practices, and policy frameworks. Enhancing agroforestry productivity requires maximizing beneficial resource interactions (e.g., available water, land, nutrients, and sunlight) while minimizing resource competition between trees and understory crops (Zhang et al., 2013; Yang et al., 2019). This requires careful design around tree species, crop types, planting patterns (spacing and canopy cover), and management practices (e.g., mowing, fertilization, and irrigation). All of them needs targeted education initiatives, hands-on training, and knowledge-sharing networks, to equip farmers with the necessary knowledge and skills for successful agroforestry implementation. governments and non-state actors must persist in research, policy formulation, and program development to overcome key barriers and enhance enabling conditions. These efforts should focus on securing land tenure rights, expanding access to technical training and knowledge, improving credit availability and short-term financing, fostering market development, and addressing inefficiencies caused by market failures and misaligned incentives (Shyamsundar et al., 2022; Schulte et al., 2022). Decades of research have demonstrated the multifunctionality of agroforestry, highlighting its role in climate change mitigation while simultaneously enhancing agricultural livelihoods and sustainability. Beyond agroforestry, a wide range of cropland transition strategies grounded in multifunctionality or circular economy principles (e.g., agrivoltaics, aquaponics, and pollinator-friendly farming) have been increasingly proposed. Each of these approaches carries distinct advantages, such as high decarbonization potential or economic returns, as well as limitations (e.g., trade-offs with biodiversity). Realizing these transition strategies’ full potential in practice requires extensive and prioritized scientific efforts to comprehensively understand the biophysical and economic constraints that shape agroforestry systems.
